# 9-Benzamido­acridinium chloride

**DOI:** 10.1107/S1600536809039439

**Published:** 2009-10-03

**Authors:** Kun Huang, Kun-Ying Liu, Da-Bin Qin

**Affiliations:** a School of Chemistry and Chemical Engineering, China West Normal University, Nanchong 637002, People’s Republic of China

## Abstract

In the title compound, C_20_H_15_N_2_O^+^·Cl^−^, the dihedral angle between the fused-ring system and the benzene ring is 63.10 (7)°. In the crystal, N—H⋯Cl hydrogen bonds link the components and aromatic π–π stacking [shortest centroid–centroid distance = 3.6421 (12) Å] occurs.

## Related literature

For background to acridine derivatives, see: Antonini (2002 ▶); Carvalho *et al.* (2005 ▶). For the synthesis, see: He *et al.* (2008 ▶); Chandregowda *et al.* (2009 ▶). For related structures, see: Sikorski *et al.* (2007 ▶, 2008 ▶); Trzybiníski *et al.* (2009 ▶).
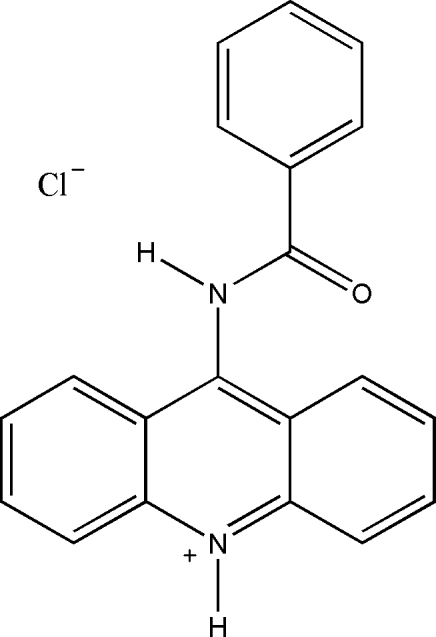


## Experimental

### 

#### Crystal data

C_20_H_15_N_2_O^+^·Cl^−^
*M* *_r_* = 334.79
Triclinic, 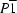

*a* = 8.9601 (17) Å
*b* = 9.0084 (17) Å
*c* = 10.8775 (18) Å
α = 79.168 (7)°
β = 65.855 (5)°
γ = 86.927 (7)°
*V* = 786.6 (2) Å^3^
*Z* = 2
Mo *K*α radiation
μ = 0.25 mm^−1^
*T* = 93 K
0.37 × 0.33 × 0.17 mm

#### Data collection

Rigaku SPIDER diffractometer
Absorption correction: multi-scan (*SADABS*; Sheldrick, 1996 ▶) *T*_min_ = 0.913, *T*_max_ = 0.959
4772 measured reflections
2955 independent reflections
2538 reflections with *I* > 2σ(*I*)
*R*_int_ = 0.020

#### Refinement

*R*[*F* ^2^ > 2σ(*F* ^2^)] = 0.037
*wR*(*F* ^2^) = 0.069
*S* = 1.00
2955 reflections
225 parameters
H atoms treated by a mixture of independent and constrained refinement
Δρ_max_ = 0.27 e Å^−3^
Δρ_min_ = −0.22 e Å^−3^

### 

Data collection: *RAPID-AUTO* (Rigaku/MSC, 2004 ▶); cell refinement: *RAPID-AUTO*; data reduction: *RAPID-AUTO*; program(s) used to solve structure: *SHELXS97* (Sheldrick, 2008 ▶); program(s) used to refine structure: *SHELXL97* (Sheldrick, 2008 ▶); molecular graphics: *SHELXTL* (Sheldrick, 2008 ▶); software used to prepare material for publication: *SHELXL97*.

## Supplementary Material

Crystal structure: contains datablocks global, I. DOI: 10.1107/S1600536809039439/hb5083sup1.cif

Structure factors: contains datablocks I. DOI: 10.1107/S1600536809039439/hb5083Isup2.hkl

Additional supplementary materials:  crystallographic information; 3D view; checkCIF report

## Figures and Tables

**Table 1:** Hydrogen-bond geometry (Å, °)

*D*—H⋯*A*	*D*—H	H⋯*A*	*D*⋯*A*	*D*—H⋯*A*
N1—H1*N*⋯Cl1^i^	0.93 (2)	2.09 (2)	3.0167 (17)	168 (8)
N2—H2*N*⋯Cl1	0.90 (2)	2.37 (2)	3.2139 (17)	154 (4)

Symmetry code: (i) 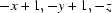.

**Fig. 1.**
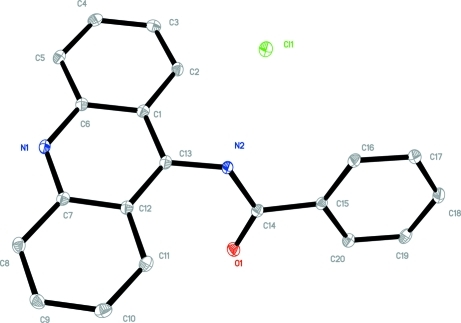
The molecular structure of the title compound, showing 30% probability displacement ellipsoids and the atomic numbering.

## supplementary crystallographic information

### Comment

Acridine derivatives which show strong antiproliferative activities on human transformed cells, have been considered as promising agents for anticancer and antiparasitic therapy (Antonini *et al.*, 2002) in recent years. Meanwhile Complexes containing acridine moiety are interesting in luminescence study (Carvalho *et al.*, 2005). Therefore they attract more and more chemists' attention. Here we report the synthesis and crystal structure of the title compound (Fig. 1).

the bond lengths and angles characterizing the geometry of the acridinium moiety are typical of acridine-based derivatives (Sikorski *et al.*, 2007, 2008). Atoms N2/C14/O1 which form dihedral angles with n1/c1/c6/*c*13/*c*12/c7 plane and *c*15 - *c*20 ring of 55.48 (1) Å and 6.82 (3) Å, respectively,are coplaner. The dihedral angle between the n1/c1/c6/*c*13/*c*12/c7 ring and *c*15 - *c*20 plane is 61.76 (6) Å.

In the crystal structure, weak Cl—H···N hydrogen bonds link the two cations and anions in ion pairs. π-π interactions between n1/c1/c6/*c*13/*c*12/c7 ring and c1-c6 ring are observed, with a *Cg*1···*Cg*2 distance of 3.6234 Å. [symmetry code: (i) –*X*,-1-Y,-*Z*] Where *Cg*1 and *Cg*2 are n1/c1/c6/*c*13/*c*12/c7 and c1-c6 centroid, respectively (Table 1).

### Experimental

The title compound was prepared according to the reported procedure of He *et al.*. (2008) & Chandregowda *et al.*. (2009). Yellow chunks of (I) were obtained by recrystallization from methanol.

### Refinement

H atoms were placed in calculated positions with C—H = 0.95–0.99 Å, & N—H = 0.9025–0.9390 Å and refined as riding with *U*_iso_(H) = 1.2*U*_eq_(C).

### Crystal data

**Table:**

C_20_H_15_N_2_O^+^·Cl^−^	*Z* = 2
*M**_r_* = 334.79	*F*(000) = 348
Triclinic, *P*1	*D*_x_ = 1.413 Mg m^−^^3^
Hall symbol: -P 1	Mo *K*α radiation, λ = 0.71073 Å
*a* = 8.9601 (17) Å	Cell parameters from 2420 reflections
*b* = 9.0084 (17) Å	θ = 3.2–27.5°
*c* = 10.8775 (18) Å	µ = 0.25 mm^−^^1^
α = 79.168 (7)°	*T* = 93 K
β = 65.855 (5)°	Chunk, yellow
γ = 86.927 (7)°	0.37 × 0.33 × 0.17 mm
*V* = 786.6 (2) Å^3^	

### Data collection

**Table:**

Rigaku SPIDER diffractometer	2955 independent reflections
Radiation source: Rotating Anode	2538 reflections with *I* > 2σ(*I*)
graphite	*R*_int_ = 0.020
ω scans	θ_max_ = 26.0°, θ_min_ = 3.2°
Absorption correction: multi-scan (*SADABS*; Sheldrick, 1996)	*h* = −8→11
*T*_min_ = 0.913, *T*_max_ = 0.959	*k* = −10→11
4772 measured reflections	*l* = −12→13

### Refinement

**Table:**

Refinement on *F*^2^	Primary atom site location: structure-invariant direct methods
Least-squares matrix: full	Secondary atom site location: difference Fourier map
*R*[*F*^2^ > 2σ(*F*^2^)] = 0.037	Hydrogen site location: inferred from neighbouring sites
*wR*(*F*^2^) = 0.069	H atoms treated by a mixture of independent and constrained refinement
*S* = 1.00	*w* = 1/[σ^2^(*F*_o_^2^) + (0.0136*P*)^2^ + 0.516*P*] where *P* = (*F*_o_^2^ + 2*F*_c_^2^)/3
2955 reflections	(Δ/σ)_max_ < 0.001
225 parameters	Δρ_max_ = 0.27 e Å^−^^3^
0 restraints	Δρ_min_ = −0.22 e Å^−^^3^

### Special details

**Table:**

Geometry. All e.s.d.'s (except the e.s.d. in the dihedral angle between two l.s. planes) are estimated using the full covariance matrix. The cell e.s.d.'s are taken into account individually in the estimation of e.s.d.'s in distances, angles and torsion angles; correlations between e.s.d.'s in cell parameters are only used when they are defined by crystal symmetry. An approximate (isotropic) treatment of cell e.s.d.'s is used for estimating e.s.d.'s involving l.s. planes.
Refinement. Refinement of *F*^2^ against ALL reflections. The weighted *R*-factor *wR* and goodness of fit *S* are based on *F*^2^, conventional *R*-factors *R* are based on *F*, with *F* set to zero for negative *F*^2^. The threshold expression of *F*^2^ > σ(*F*^2^) is used only for calculating *R*-factors(gt) *etc*. and is not relevant to the choice of reflections for refinement. *R*-factors based on *F*^2^ are statistically about twice as large as those based on *F*, and *R*- factors based on ALL data will be even larger.

### Fractional atomic coordinates and isotropic or equivalent isotropic displacement parameters (Å^2^)

**Table:**

	*x*	*y*	*z*	*U*_iso_*/*U*_eq_	
Cl1	0.78153 (5)	0.85506 (5)	−0.03376 (4)	0.01909 (12)	
O1	0.39684 (14)	0.63975 (13)	0.49853 (12)	0.0187 (3)	
N1	0.34182 (17)	0.35116 (16)	0.16256 (14)	0.0143 (3)	
N2	0.54610 (18)	0.68607 (16)	0.26770 (15)	0.0153 (3)	
C1	0.5768 (2)	0.46569 (18)	0.16353 (17)	0.0139 (4)	
C2	0.7475 (2)	0.45912 (19)	0.13000 (17)	0.0161 (4)	
H2	0.7988	0.5317	0.1541	0.019*	
C3	0.8384 (2)	0.34982 (19)	0.06364 (18)	0.0178 (4)	
H3	0.9524	0.3469	0.0422	0.021*	
C4	0.7652 (2)	0.2403 (2)	0.02605 (18)	0.0185 (4)	
H4	0.8306	0.1652	−0.0205	0.022*	
C5	0.6021 (2)	0.24183 (19)	0.05601 (17)	0.0165 (4)	
H5	0.5538	0.1687	0.0301	0.020*	
C6	0.5053 (2)	0.35355 (18)	0.12623 (17)	0.0142 (4)	
C7	0.2393 (2)	0.44990 (18)	0.23448 (17)	0.0140 (4)	
C8	0.0696 (2)	0.4369 (2)	0.27045 (18)	0.0176 (4)	
H8	0.0268	0.3567	0.2478	0.021*	
C9	−0.0322 (2)	0.5401 (2)	0.33778 (18)	0.0194 (4)	
H9	−0.1468	0.5297	0.3649	0.023*	
C10	0.0313 (2)	0.6631 (2)	0.36779 (18)	0.0197 (4)	
H10	−0.0406	0.7372	0.4106	0.024*	
C11	0.1935 (2)	0.67615 (19)	0.33612 (18)	0.0172 (4)	
H11	0.2338	0.7592	0.3571	0.021*	
C12	0.3043 (2)	0.56678 (18)	0.27180 (17)	0.0141 (4)	
C13	0.4735 (2)	0.57227 (18)	0.23643 (17)	0.0139 (4)	
C14	0.5083 (2)	0.70877 (18)	0.39917 (18)	0.0145 (4)	
C15	0.6172 (2)	0.82148 (18)	0.41153 (17)	0.0139 (4)	
C16	0.7367 (2)	0.91166 (19)	0.30042 (18)	0.0175 (4)	
H16	0.7528	0.9047	0.2098	0.021*	
C17	0.8326 (2)	1.01193 (19)	0.32150 (19)	0.0198 (4)	
H17	0.9140	1.0735	0.2453	0.024*	
C18	0.8099 (2)	1.02228 (19)	0.45330 (19)	0.0187 (4)	
H18	0.8765	1.0902	0.4674	0.022*	
C19	0.6903 (2)	0.93372 (19)	0.56455 (19)	0.0190 (4)	
H19	0.6746	0.9412	0.6550	0.023*	
C20	0.5936 (2)	0.83416 (19)	0.54403 (18)	0.0165 (4)	
H20	0.5109	0.7743	0.6206	0.020*	
H1N	0.298 (2)	0.278 (2)	0.1340 (19)	0.023 (5)*	
H2N	0.633 (2)	0.736 (2)	0.197 (2)	0.024 (5)*	

### Atomic displacement parameters (Å^2^)

**Table:**

	*U*^11^	*U*^22^	*U*^33^	*U*^12^	*U*^13^	*U*^23^
Cl1	0.0200 (2)	0.0198 (2)	0.0173 (2)	−0.00404 (17)	−0.00573 (19)	−0.00612 (18)
O1	0.0190 (7)	0.0196 (6)	0.0160 (7)	−0.0045 (5)	−0.0056 (6)	−0.0020 (5)
N1	0.0175 (8)	0.0134 (7)	0.0137 (8)	−0.0022 (6)	−0.0077 (6)	−0.0023 (6)
N2	0.0160 (8)	0.0165 (8)	0.0128 (8)	−0.0038 (6)	−0.0040 (7)	−0.0045 (6)
C1	0.0177 (9)	0.0131 (8)	0.0103 (9)	−0.0006 (7)	−0.0060 (7)	−0.0001 (7)
C2	0.0183 (9)	0.0165 (9)	0.0147 (9)	−0.0034 (7)	−0.0083 (8)	−0.0011 (7)
C3	0.0164 (10)	0.0183 (9)	0.0166 (10)	0.0015 (7)	−0.0058 (8)	−0.0008 (8)
C4	0.0230 (10)	0.0168 (9)	0.0139 (9)	0.0029 (7)	−0.0062 (8)	−0.0027 (7)
C5	0.0228 (10)	0.0141 (9)	0.0143 (9)	−0.0002 (7)	−0.0090 (8)	−0.0029 (7)
C6	0.0175 (10)	0.0143 (8)	0.0102 (9)	−0.0022 (7)	−0.0061 (7)	0.0010 (7)
C7	0.0175 (9)	0.0139 (8)	0.0104 (9)	−0.0008 (7)	−0.0061 (7)	−0.0004 (7)
C8	0.0194 (10)	0.0198 (9)	0.0155 (9)	−0.0030 (7)	−0.0094 (8)	−0.0018 (8)
C9	0.0144 (9)	0.0277 (10)	0.0166 (9)	−0.0007 (8)	−0.0074 (8)	−0.0028 (8)
C10	0.0203 (10)	0.0234 (10)	0.0159 (10)	0.0054 (8)	−0.0077 (8)	−0.0055 (8)
C11	0.0215 (10)	0.0169 (9)	0.0156 (9)	0.0009 (7)	−0.0096 (8)	−0.0039 (8)
C12	0.0170 (9)	0.0143 (8)	0.0109 (9)	−0.0013 (7)	−0.0060 (7)	−0.0007 (7)
C13	0.0188 (10)	0.0126 (8)	0.0101 (8)	−0.0030 (7)	−0.0064 (7)	0.0004 (7)
C14	0.0159 (9)	0.0130 (8)	0.0166 (9)	0.0033 (7)	−0.0085 (8)	−0.0040 (7)
C15	0.0148 (9)	0.0121 (8)	0.0169 (9)	0.0024 (7)	−0.0078 (8)	−0.0045 (7)
C16	0.0202 (10)	0.0186 (9)	0.0150 (9)	−0.0005 (7)	−0.0070 (8)	−0.0058 (8)
C17	0.0201 (10)	0.0181 (9)	0.0187 (10)	−0.0039 (8)	−0.0044 (8)	−0.0042 (8)
C18	0.0200 (10)	0.0164 (9)	0.0238 (10)	0.0003 (7)	−0.0118 (8)	−0.0064 (8)
C19	0.0261 (10)	0.0183 (9)	0.0171 (10)	0.0026 (8)	−0.0123 (8)	−0.0063 (8)
C20	0.0205 (10)	0.0141 (8)	0.0150 (9)	−0.0002 (7)	−0.0077 (8)	−0.0015 (7)

### Geometric parameters (Å, °)

**Table:**

O1—C14	1.218 (2)	C8—H8	0.9500
N1—C6	1.352 (2)	C9—C10	1.419 (2)
N1—C7	1.354 (2)	C9—H9	0.9500
N1—H1N	0.939 (19)	C10—C11	1.356 (2)
N2—C14	1.381 (2)	C10—H10	0.9500
N2—C13	1.405 (2)	C11—C12	1.425 (2)
N2—H2N	0.902 (19)	C11—H11	0.9500
C1—C13	1.414 (2)	C12—C13	1.404 (2)
C1—C2	1.421 (2)	C14—C15	1.505 (2)
C1—C6	1.425 (2)	C15—C16	1.390 (2)
C2—C3	1.362 (2)	C15—C20	1.395 (2)
C2—H2	0.9500	C16—C17	1.389 (2)
C3—C4	1.418 (2)	C16—H16	0.9500
C3—H3	0.9500	C17—C18	1.384 (2)
C4—C5	1.360 (2)	C17—H17	0.9500
C4—H4	0.9500	C18—C19	1.385 (2)
C5—C6	1.415 (2)	C18—H18	0.9500
C5—H5	0.9500	C19—C20	1.387 (2)
C7—C8	1.412 (2)	C19—H19	0.9500
C7—C12	1.426 (2)	C20—H20	0.9500
C8—C9	1.361 (2)		
			
C6—N1—C7	123.57 (14)	C11—C10—C9	120.77 (16)
C6—N1—H1N	117.6 (11)	C11—C10—H10	119.6
C7—N1—H1N	118.8 (11)	C9—C10—H10	119.6
C14—N2—C13	124.05 (15)	C10—C11—C12	120.82 (16)
C14—N2—H2N	120.0 (12)	C10—C11—H11	119.6
C13—N2—H2N	115.3 (12)	C12—C11—H11	119.6
C13—C1—C2	123.94 (15)	C13—C12—C11	124.12 (15)
C13—C1—C6	118.42 (15)	C13—C12—C7	118.46 (15)
C2—C1—C6	117.58 (15)	C11—C12—C7	117.32 (15)
C3—C2—C1	120.64 (16)	C12—C13—N2	121.44 (15)
C3—C2—H2	119.7	C12—C13—C1	120.60 (15)
C1—C2—H2	119.7	N2—C13—C1	117.93 (15)
C2—C3—C4	120.88 (17)	O1—C14—N2	122.22 (15)
C2—C3—H3	119.6	O1—C14—C15	122.34 (15)
C4—C3—H3	119.6	N2—C14—C15	115.42 (15)
C5—C4—C3	120.67 (16)	C16—C15—C20	119.27 (15)
C5—C4—H4	119.7	C16—C15—C14	124.14 (15)
C3—C4—H4	119.7	C20—C15—C14	116.59 (15)
C4—C5—C6	119.29 (16)	C17—C16—C15	120.21 (16)
C4—C5—H5	120.4	C17—C16—H16	119.9
C6—C5—H5	120.4	C15—C16—H16	119.9
N1—C6—C5	119.64 (15)	C18—C17—C16	120.14 (17)
N1—C6—C1	119.40 (15)	C18—C17—H17	119.9
C5—C6—C1	120.93 (16)	C16—C17—H17	119.9
N1—C7—C8	119.67 (15)	C17—C18—C19	120.02 (16)
N1—C7—C12	119.52 (15)	C17—C18—H18	120.0
C8—C7—C12	120.81 (15)	C19—C18—H18	120.0
C9—C8—C7	119.44 (16)	C18—C19—C20	120.04 (16)
C9—C8—H8	120.3	C18—C19—H19	120.0
C7—C8—H8	120.3	C20—C19—H19	120.0
C8—C9—C10	120.61 (17)	C19—C20—C15	120.31 (16)
C8—C9—H9	119.7	C19—C20—H20	119.8
C10—C9—H9	119.7	C15—C20—H20	119.8
			
C13—C1—C2—C3	177.69 (17)	C8—C7—C12—C11	−5.1 (2)
C6—C1—C2—C3	0.5 (3)	C11—C12—C13—N2	2.5 (3)
C1—C2—C3—C4	0.2 (3)	C7—C12—C13—N2	178.76 (16)
C2—C3—C4—C5	−0.2 (3)	C11—C12—C13—C1	−175.20 (17)
C3—C4—C5—C6	−0.4 (3)	C7—C12—C13—C1	1.0 (2)
C7—N1—C6—C5	177.34 (16)	C14—N2—C13—C12	61.0 (2)
C7—N1—C6—C1	−0.5 (2)	C14—N2—C13—C1	−121.22 (18)
C4—C5—C6—N1	−176.64 (16)	C2—C1—C13—C12	−176.74 (16)
C4—C5—C6—C1	1.2 (3)	C6—C1—C13—C12	0.4 (2)
C13—C1—C6—N1	−0.7 (2)	C2—C1—C13—N2	5.4 (3)
C2—C1—C6—N1	176.61 (16)	C6—C1—C13—N2	−177.42 (15)
C13—C1—C6—C5	−178.53 (16)	C13—N2—C14—O1	−7.0 (3)
C2—C1—C6—C5	−1.2 (2)	C13—N2—C14—C15	171.42 (15)
C6—N1—C7—C8	−178.65 (16)	O1—C14—C15—C16	−174.41 (17)
C6—N1—C7—C12	2.0 (3)	N2—C14—C15—C16	7.1 (2)
N1—C7—C8—C9	−177.30 (17)	O1—C14—C15—C20	5.4 (2)
C12—C7—C8—C9	2.1 (3)	N2—C14—C15—C20	−173.03 (15)
C7—C8—C9—C10	2.2 (3)	C20—C15—C16—C17	0.7 (3)
C8—C9—C10—C11	−3.3 (3)	C14—C15—C16—C17	−179.45 (16)
C9—C10—C11—C12	0.1 (3)	C15—C16—C17—C18	0.2 (3)
C10—C11—C12—C13	−179.75 (17)	C16—C17—C18—C19	−0.7 (3)
C10—C11—C12—C7	4.0 (3)	C17—C18—C19—C20	0.2 (3)
N1—C7—C12—C13	−2.2 (2)	C18—C19—C20—C15	0.6 (3)
C8—C7—C12—C13	178.44 (16)	C16—C15—C20—C19	−1.1 (3)
N1—C7—C12—C11	174.28 (15)	C14—C15—C20—C19	179.04 (16)

### Hydrogen-bond geometry (Å, °)

**Table:**

*D*—H···*A*	*D*—H	H···*A*	*D*···*A*	*D*—H···*A*
N1—H1N···Cl1^i^	0.93 (2)	2.09 (2)	3.0167 (17)	168 (8)
N2—H2N···Cl1	0.90 (2)	2.37 (2)	3.2139 (17)	154 (4)

Symmetry codes: (i) −*x*+1, −*y*+1, −*z*.
